# Aortic aneurysm rupture as a rare complication of granulomatosis with polyangiitis: a case report

**DOI:** 10.1186/1752-1947-7-202

**Published:** 2013-07-26

**Authors:** Nobuo Ohta, Takayoshi Waki, Shigeru Fukase, Yusuke Suzuki, Kazuya Kurakami, Masaru Aoyagi, Seiji Kakehata

**Affiliations:** 1 Department of Otolaryngology, Yamagata University Faculty of Medicine, 2-2-2 Iida-nishi, Yamagata, 990-23, Japan

**Keywords:** Aortic aneurysm, Aortitis, Wegener’s granulomatosis

## Abstract

**Introduction:**

Granulomatosis with polyangiitis is characterized by systemic inflammation of medium and small blood vessels. Aortic involvement in granulomatosis with polyangiitis is extremely rare. As far as we know this is the first reported case of successful treatment in a patient with granulomatosis with polyangiitis complicated with aortic aneurysm rupture.

**Case presentation:**

We describe a case of granulomatosis with polyangiitis in a 38-year-old Japanese man who developed an aortic aneurysm rupture 22 years after disease onset. The patient was operated on and a J-graft was inserted. He recovered uneventfully.

**Conclusion:**

Recommendations in regard to, and consideration of, aortic involvement should be kept in mind in the long-term careful follow up of granulomatosis with polyangiitis.

## Introduction

Granulomatosis with polyangiitis (GPA) is characterized by a systemic necrotizing vasculitis affecting the small and medium blood vessels. Typically, the upper and lower respiratory tract and often the kidneys are involved [1-4]. Aortic involvement in GPA is extremely rare [5-12], but because of the potential life-threatening risk of rupture it is important to recognize aortic or other large vessel involvement (Table 1). We present a case of GPA in a patient with rupture of a thoracic aortic aneurysm as a complication.

**Table 1:** Cases of aortic involvement in granulomatosis with polyangiitis

***Ref.***	***Age/gender (years)***	***Affected site***	***Duration of complaint***	***Treatment***	***Rupture***	***Outcome***
[5]	53/F	3.8cm	5 months	Conservative	No	Good
[6]	45/M	No AAA	1 week	Surgery	No	Good
[7]	33/F	3.2cm	3 weeks	Surgery	No	Good
[8]	42/M	3.2cm	1 week	Surgery	No	Good
[9]	63/M	4cm	1 week	Surgery	No	Good
[10]	50/F	AAA	1 week	Conservative	Yes	Death
[11]	43/M	3.5cm	1 week	Surgery	No	Good
[12]	29/M	AAA	NS	Conservative	No	Good

*AAA* abdominal aortic aneurysm, *F* female, *M* male, *NS* not stated.

## Case presentation

**Figure 1:**
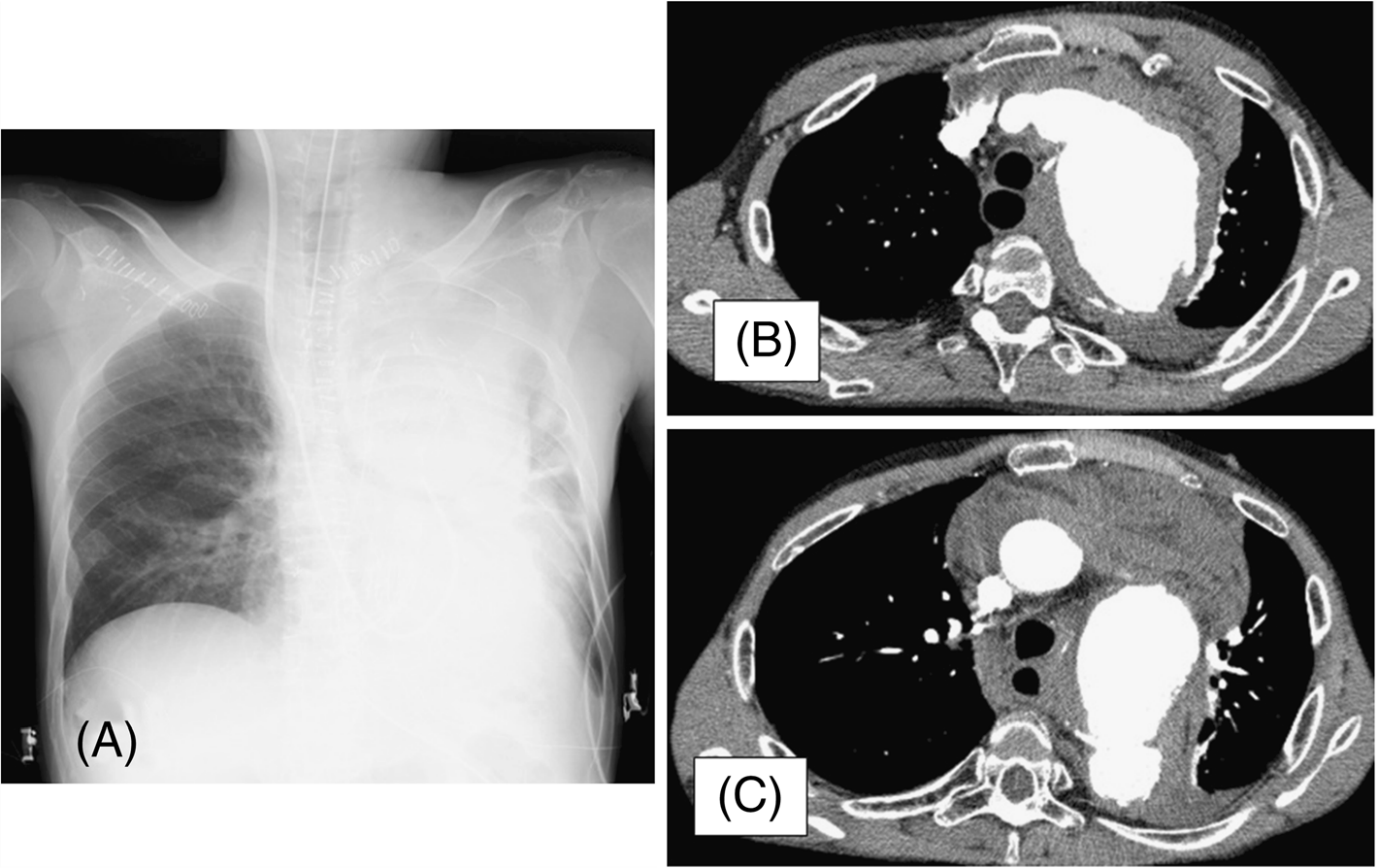
**Chest radiograph and computed tomography scan of patient with GPA. (A)** Chest radiograph of a patient with a very large aneurysm of the aortic arch. Evident are marked widening of the mediastinum and aortic contour. **(B)** and **(C)** Axial contrast-enhanced computed tomography scan of the thorax revealing ruptured aortic aneurysm and collapsed left lung.

**Figure 2:**
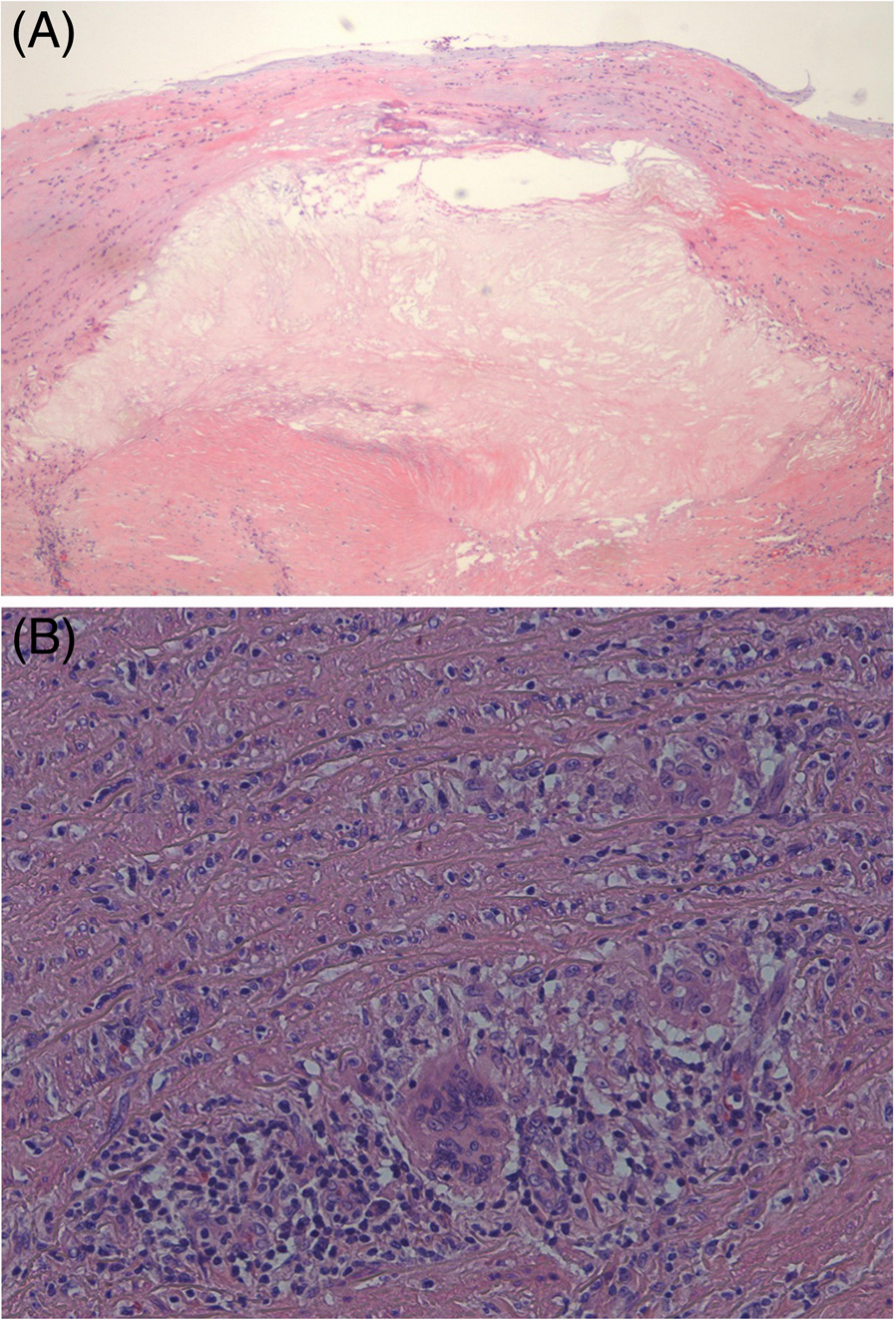
**Histpathological findings of aortic aneurysm from patient with GPA. (A)** Histologic section showing considerable arteriosclerosis in the intima and media, with cholesterol clefts. There is intimal thickening with reduplication of the internal elastic lamina. The media is thickened, and the medial smooth muscle cells are replaced by pink hyaline material (hematoxylin and eosin, original magnification ×100). **(B)** Arteritis with giant-cell formation and lymphocyte and plasma cell infiltration around the vasa vasorum in the media is evident. The aortic media shows patchy infiltration by lymphocytes (plasma cells), with early disruption of the media (hematoxylin and eosin, original magnification ×270).

A 38-year-old Japanese man was admitted to our hospital with the chief complaint of back pain and loss of consciousness. He had been diagnosed with GPA on the basis of the presence of inflammatory nasopharyngeal lesions and glomerulonephritis 22 years ago. At the time, histopathologic examination of a biopsy specimen from the nasal cavity had shown necrotizing giant-cell granulomatous inflammation and a necrotizing granulomatous vasculitis. A serum sample was positive by indirect immunofluorescence for anti-neutrophil cytoplasmic antibodies (×128) (i.e., anti-neutrophil cytoplasmic antibodies were detected in 128 times the diluted serum sample). With the exception of three recurrences, the GPA had remained in remission with treatment with prednisolone (15mg/day). At this latest admission, the patient had chest pain on physical examination. Laboratory tests showed the following: leukocyte count, 15.15 × 10^9^/L; hemoglobin, 10.8mmol/L; C-reactive protein, 1.36mg/dL; and stable serum creatinine, 1.31mg/dL. A chest X-ray showed lateral shift of the left lung (Figure 1A). A thoracic computed tomography (CT) scan showed enlargement of the wall of the thoracic aorta, which was surrounded by a hypo-dense rim, and rupture of a dissected aortic aneurysm (Figure 1B and 1C). The patient underwent emergency surgery. Rupture of a 70mm length of the distal arch of the aorta was confirmed during surgery, and a J-graft was inserted. Culture for syphilis was negative. The aortic aneurysm was embedded in an area of tissue inflammation. Histopathologic examination showed a necrotizing inflammation of the aortic wall and a necrotizing granulomatous vasculitis (Figure 2). The diagnosis of aortic rupture as a complication of GPA was made on pathological findings. Postoperatively, the patient recovered uneventfully and was discharged on the 21st postoperative day. Treatment was continued and consisted of prednisolone (15mg/day). After 6 months, a positron emission tomography scan showed no new signs of vascular inflammation or other aneurysm.

## Discussion

Although GPA is characterized by a systemic necrotizing inflammation of the medium and small arteries, aortic involvement is very uncommon. In general, aortic involvement is more frequently seen in large-vessel vasculitides such as those seen in giant cell arteritis and Takayasu’s arteritis [1-4]. GPA can affect the respiratory and renal tracts. It can also affect the eyes, skin, and peripheral nerves. Non-specific systemic symptoms are common. GPA causes upper respiratory tract disease in >90% of cases and causes sinusitis, nasal crusting, bleeding, and obstruction, and collapse of the nasal bridge [5-8]. It can also cause otitis media and tracheal stenosis. When the lungs are affected, it may present as cough, hemoptysis, and dyspnea. Renal involvement may manifest as hematuria and proteinuria and can lead to renal failure. Ophthalmological manifestations include subconjunctival hemorrhage, scleritis, uveitis, keratitis, proptosis, or ocular muscle paralysis due to retro-orbital inflammation. Serology is positive for anti-proteinase 3 (anti-neutrophil cytoplasmic antibody), which is highly specific for GPA. The differential diagnosis of GPA includes polyarteritis nodosa, Churg–Strauss syndrome, Henoch–Schönlein purpura, temporal arteritis, and Takayasu’s arteritis. The main treatment is immunosuppression and steroid therapy. A combination of cyclophosphamide and prednisone is effective. Our patient had been receiving prednisolone (15mg/day) for 11 years (since his diagnosis) and had remained negative for anti-neutrophil cytoplasmic antibody until he presented with the aneurysm.

Aortic aneurysm is a very rare complication of GPA. To the best of our knowledge, nine other cases of aneurysm of large vessels as a result of GPA have been described [5-15] and this is the first reported case of successful treatment in a patient with GPA complicated with aortic aneurysm rupture. In five of the cases, the patients were operated on and surgical tissue biopsies showed signs of vasculitis indicative of GPA. In one case, the patient was treated with steroids and cyclophosphamide, but medical treatment could not prevent aortic dissection and the patient died from rupture of the dissection. In three cases, the patients were treated medically with drugs that included methylprednisolone and trimethoprim-sulfamethoxazole. CT is an extremely accurate tool for both diagnosis and sizing of aortic aneurysms [5-9]. A longitudinal cohort study of GPA patients evaluated at the U.S. National Institutes of Health between 1968 and 1992 demonstrated that 91% experienced clinical improvement and 75% entered complete remission when treated with cyclophosphamide and methylprednisolone [7]. A meta-analysis revealed that pulse cyclophosphamide is less toxic than continuous cyclophosphamide but may be associated with a higher relapse rate [5].

In summary, chest pain occurring during a GPA flare may result from aortic involvement and may cause a life-threatening aneurysm rupture for which surgical treatment is indicated. Therefore, aortic involvement should be kept in mind as a potential fatal complication of GPA.

## Conclusion

We presented an extremely rare case of thoracic aortic aneurysm rupture as a complication of GPA. GPA should be included in the workup of large-vessel vasculitis, which can give rise to potentially life-threatening periaortic inflammation.

## Consent

Written informed consent was obtained from the patient for publication of this manuscript and accompanying images. A copy of the written consent is available for review by the Editor-in-Chief of this journal.

## Competing interests

The authors declare that they have no competing interests.

## Authors’ contributions

NO used all of the data available and wrote the majority of this report. SF was the main consultant surgeon involved in the management of this patient. YS and SK supplied the principles of surgical information in this article. KK and MA saw the patient in hospital and contributed the case history notes used in this report. WT reported and provided us with the histopathological findings and slides. All authors read and approved the final manuscript.
